# Reduced Luteinizing Hormone Induction Following Estrogen and Progesterone Priming in Female-to-Male Transsexuals

**DOI:** 10.3389/fendo.2018.00212

**Published:** 2018-05-07

**Authors:** Toshiya Funabashi, Hideya Sakakibara, Fumiki Hirahara, Fukuko Kimura

**Affiliations:** ^1^Department of Physiology, Yokohama City University School of Medicine, Yokohama, Japan; ^2^Department of Physiology, St. Marianna University School of Medicine, Kawasaki, Japan; ^3^Department of Obstetrics and Gynecology, Yokohama City University School of Medicine, Yokohama, Japan; ^4^Department of Gynecology, Yokohama City University Medical Center, Yokohama, Japan; ^5^Tanaka Clinic Yokohama-Koen, Yokohama, Japan

**Keywords:** transsexual, gender dysphoria, gender identity, estrogen, progesterone, luteinizing hormone, bed nucleus, human

## Abstract

Anatomical studies have suggested that one of the brain structures involved in gender identity is the bed nucleus of the stria terminalis, though this brain structure is probably not the only one to control gender identity. We hypothesized that, if this brain area also affected gonadotropin secretion in humans, transsexual individuals might produce different gonadotropin levels in response to exogenous stimulation. In the present study, we examined whether estrogen combined with progesterone might lead to a change in luteinizing hormone (LH) secretion in female-to-male (FTM) transsexual individuals. We studied female control subjects (*n* = 9), FTM transsexual subjects (*n* = 12), and male-to-female (MTF) transsexual subjects (*n* = 8). Ethinyl estradiol (50 μg/tablet) was administered orally, twice a day, for five consecutive days. After the first blood sampling, progesterone (12.5 mg) was injected intramuscularly. Plasma LH was measured with an immunoradiometric assay. The combination of estrogen and progesterone resulted in increased LH secretion in female control subjects and in MTF subjects, but this increase appeared to be attenuated in FTM transsexual subjects. In fact, the %LH response was significantly reduced in FTM subjects (*P* < 0.05), but not in MTF subjects (*P* > 0.5), compared to female control subjects. In addition, the peak time after progesterone injection was significantly delayed in FTM subjects (*P* < 0.05), but not in MTF subjects (*P* > 0.5), compared to female control subjects. We then compared subjects according to whether the combination of estrogen and progesterone had a positive (more than 200% increase) or negative (less than 200% increase) effect on LH secretion. A χ^2^ analysis revealed significantly different (*P* < 0.05) effects on LH secretion between female controls (positive *n* = 7, negative *n* = 2) and FTM transsexual subjects (positive *n* = 4, negative *n* = 8), but not between female controls and MTF transsexual subjects (positive *n* = 7, negative *n* = 1). Thus, LH secretion in response to estrogen- and progesterone priming was attenuated in FTM subjects, but not in MTF subjects, compared to control females. This finding suggested that the brain area related to gender identity in morphological studies might also be involved in the LH secretory response in humans. Thus, altered brain morphology might be correlated to altered function in FTM transsexuals.

## Introduction

It is generally agreed that the rodent central nervous system is sexually differentiated. This includes the hypothalamus and the anterior pituitary, which control luteinizing hormone (LH) secretion. Female rodents, but not males, exhibit a surge of LH secretion that drives ovulation, which results from a “positive feedback” effect of estrogen (1). Androgen exposure during the neonatal period is thought to be critical for the development of sexual differentiation (2–5). Thus, the presence of a sex difference, or sexual dimorphism, is found in many brain structures, due to the organizational effects of sex steroids, which produce male- or female-like morphology.

The bed nucleus of the stria terminalis (BST) is one of several structures that show sexually dimorphic regions (6, 7). Moreover, neuronal fibers that project from the BST to various brain regions (8–10) are involved in many different types of motivational behaviors (11–13). In addition, the BST was shown to be involved in gonadotropin secretion (14–17). Indeed, estrogen-binding cells were found in the BST (18), where gonadal steroid receptors were expressed (19–21). The rat lateral subdivision of the BST is homologous to the human central BST (22, 23), which is thought be involved in gender identity in humans.

Gender identity disorder, or gender dysphoria (GD), is defined as a strong gender identification with the opposite sex; for example, female-to-male (FTM) transsexuals have a strong male gender identity, and thus, they want to reassign their physical sex (24–26). Although GD is a highly complex clinical situation, its etiology has been described by several environmental, genetic, and anatomical theories (27–30). Currently, there is morphological evidence that, in humans, the BST is related to gender identity (31–33). Studies have shown that the number of neurons in the BST (34) and the central BST size (35) in male-to-female (MTF) transsexual individuals corresponded to the respective norms for biological females. Conversely, FTM transsexual individuals exhibited a BST size that corresponded to the norm for biological males (35). It is unknown whether these structural features are causally related to sexual identity or whether they are indirectly correlated.

Taken together, findings from previous studies have led to the hypothesis that, if the same area of the central nervous system that controls LH secretions is also involved in gender identity, or conversely, if the causal factor(s) (as yet undetermined) that affects brain structure in GD can also account for LH secretion, then the LH secretory profile in subjects with GD might be different from the typical profile expected for a given sex. Gooren and colleagues studied this hypothesis in detail (36, 37) and concluded that the neuroendocrine regulation of LH secretion did not differ between transsexual and non-transsexual individuals, either males or females (24, 28). This result may be reasonable, since the neuroendocrine mechanism that regulates the cyclic release (surge) of LH in primates is not thought to be different between the sexes (5, 38). Indeed, estrogen was equally capable of inducing surge-like LH secretions in male rhesus monkeys (5, 38–40) and female monkeys (1). In humans, it was reported that a similar surge-like LH secretion could be induced by estrogen in gonadectomized men (41, 42).

However, androgen exposure during the neonatal period, which is essential for brain differentiation in rodents (2, 3, 38), may alter the LH secretory response later in life, even in female primates (39, 43–46). It was reported that, in estrogen-primed ovariectomized female rhesus monkeys, progesterone had a stimulatory effect on LH secretion (47, 48). Importantly, this stimulatory effect was unremarkable in orchidectomized male rhesus macaques pretreated with 17-β estradiol (49). Although one report suggested that a surge-like LH secretion could be induced in men with progesterone after estrogen priming (50), the response was not directly compared with responses in women subjects; thus, the amplitude of the effect was unclear. Therefore, we hypothesized that estrogen combined with progesterone might lead to a change in LH secretion in patients with GD.

## Subjects and Methods

### Subjects

For the present study, we recruited healthy Japanese volunteers who had undergone either FTM or MTF transsexual procedures. All FTM subjects were diagnosed with GD by psychiatrists, independent of the present study. All MTF subjects self-reported that they were bilaterally orchidectomized. Although, ideally, MTF subjects should be compared to male control subjects with bilateral orchidectomies, we could not recruit these control subjects. Thus, we compared the MTF transsexual data to female control data. One month before the sampling experiment, MTF subjects were asked to stop their cross-gender hormone treatments. All subjects self-reported their sexual orientation.

### Treatment

Ethinyl estradiol (50 µg) tablets were administered to all subjects orally twice per day (100 µg total per day) for five consecutive days (days 3 to 7). Day 1 of the experiment was the day that menstruation began in female and FTM subjects. In previous studies, this dose of estrogen alone did not induce LH secretion, but when combined with progesterone, it induced a surge-like LH secretion (51–53). The estrogen treatment period was chosen, based on previous reports (51–53) and on our previous experience with rats (54). Blood samples were drawn on day 7. After the blood sample was taken, progesterone (12.5 mg) was injected at the University Hospital, between 0830 and 0930 h. Then, sequential blood samples (1.0–1.5 ml each) were collected on the same day, at 1200, 1500, 1800, and 2100 h. The final blood sample was drawn at 0900 h the next day. All blood samples were drawn through a median cubital vein. Plasma was separated from the blood samples at 4°C and stored at −20°C.

### Ethics

This study was conducted in accordance with the recommendations of the Ethical Guidelines for Medical and Health Research Involving Human Subjects, established by the Ministry of Education, Culture, Sports, Science, and Technology and the Ministry of Health, Labor and Welfare. We obtained written informed consent to participate in the study from all volunteers, in accordance with the Declaration of Helsinki. The protocol was approved by the institutional Ethics Committee of the Yokohama City University School of Medicine. Subjects were interviewed to obtain data on their general health condition, sexual orientation, and menstrual cycles.

### Analytical Methods and Statistical Analyses

Plasma LH was measured with immunoradiometric assay kits (Daiichi Radioisotope Institute). The intra- and inter-assay coefficients of variation, estimated at the mean LH level of 7.6 mIU/ml, were 3.0 and 8.0%, respectively. We tested statistical significance, as appropriate, with the one-way ANOVA, followed by Fisher’s Least Significant Differences (LSD) *post hoc* comparison; with the Kruskal–Wallis test, followed by Dunn’s multiple comparison test; or with the χ^2^ test. Significance was accepted at *P* < 0.05.

## Results

### Subjects

**Figure 1:**
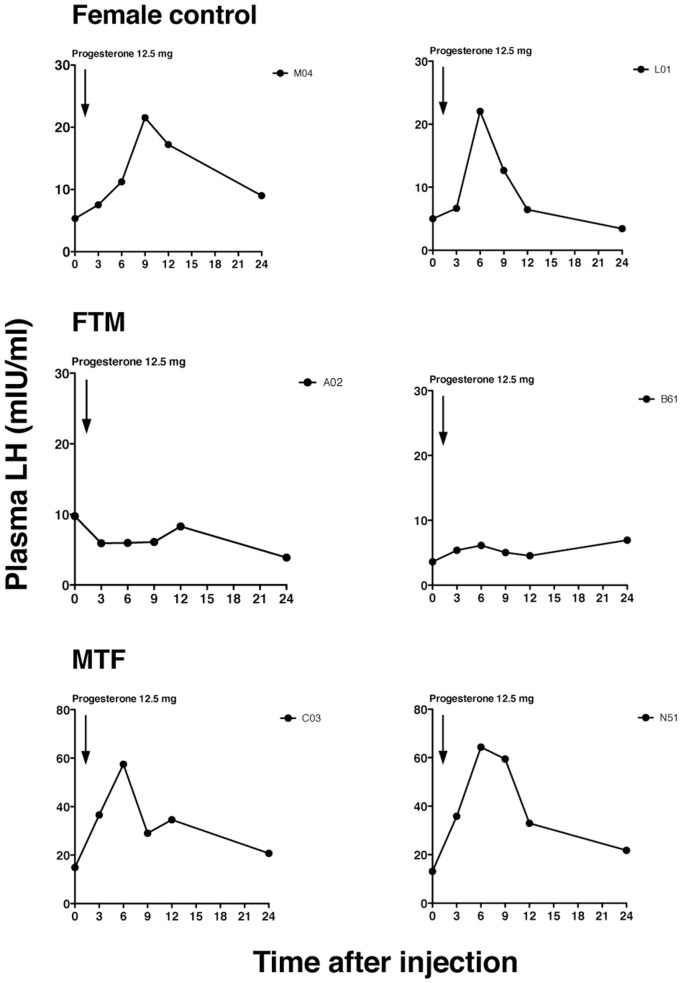
Representative luteinizing hormone (LH) secretory patterns in transsexual individuals. Profiles of the LH secretory pattern in two female controls *(upper panels)*, two female-to-male transsexual (FTM) subjects *(middle panels)*, and two male-to-female transsexual (MTF) subjects *(lower panels)*, after estrogen priming (ethinyl estradiol, 100 µg for 5 days), and before (time 0) and after the progesterone injection. Each point indicates the plasma LH level. The arrow indicates the time of progesterone injection (12.5 mg).

A total of nine female control subjects, 12 FTM subjects, and 8 MTF subjects, aged 28.7 ± 2.7, 27.4 ± 1.5, and 39.3 ± 2.3 years, respectively, participated in blood sampling experiments. We found that the baseline LH levels (before injection) were higher in MTF subjects (Figure 1) than in the other subjects. This finding suggested that no negative feedback was present, consistent with the removal of the testes. All female control and 10 FTM subjects self-reported regular menstrual cycles (regular was defined as a menstrual cycle of 25–38 days). Sexual orientations were self-reported; all female control subjects were attracted to men. All FTM subjects were attracted to women. Among the MTF subjects, one was attracted to women, four were to men, and three were attracted to both.

### Effects of Estrogen Combined With Progesterone on LH Secretion

The LH response to estrogen and progesterone was tentatively defined as positive, when LH levels were greater than 200% of the control level. This threshold was based on data from previous studies (55, 56). According to this criterion, 77.7% of control females and 33.3% of FTM subjects showed positive responses (Table 1, *P* < 0.05, χ^2^ test). In addition, seven out of eight MTF subjects displayed positive responses, a similar proportion to that observed among control females (*P* > 0.1).

**Table 1:** Luteinizing hormone (LH) response to estrogen and progesterone stimulation, in control females and transsexual subjects.

Subjects	Number	LH response^a^		*P*-value
			
		Positive	Negative	
Control	9	7	2	
Female-to-Male	12	4	8	*
Male-to-Female	8	7	1	NS

^a^The LH responses were classified as positive (more than 200% increase) or negative (less than 200% increase), compared to baseline. *P < 0.05 vs. female control subjects, based on the χ^2^ test. NS = not significant.

**Figure 2:**
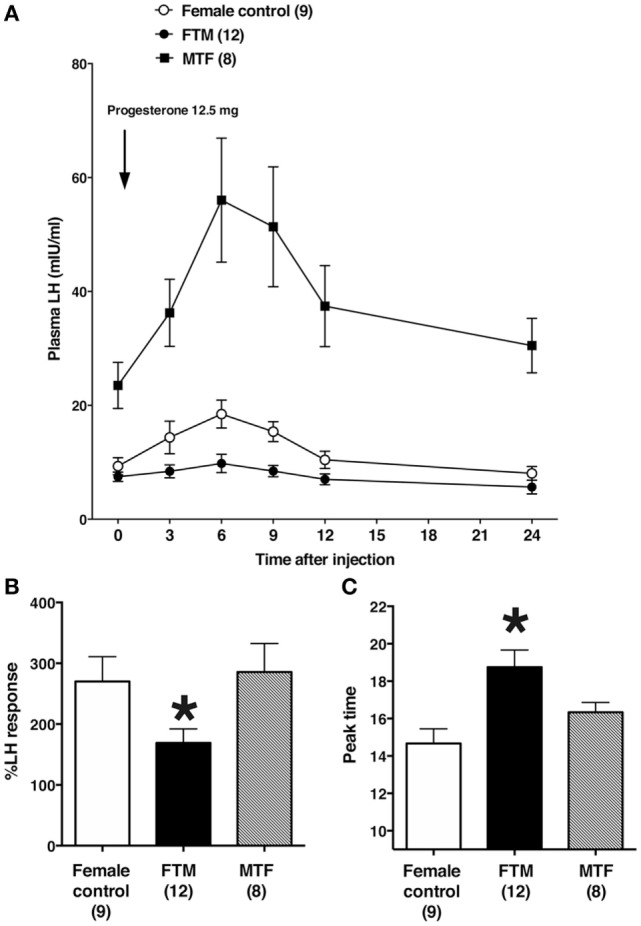
Summary of the effects of estrogen and progesterone treatment on luteinizing hormone (LH) secretion in transsexuals. **(A)** Effects on LH secretion over time in female controls (open circles), female-to-male transsexual (FTM) subjects (closed circles), and male-to-female transsexual (MTF) subjects (closed squares). Each point and vertical line indicate the mean and SE, respectively. The LH level after 5 days of estrogen is shown at time 0. The arrow indicates the time of progesterone injection (12.5 mg). **(B)** The peak %changes in plasma LH are shown after estrogen and progesterone treatment, in female controls (*open bars*), FTM transsexual subjects (*filled bars*), and MTF transsexual subjects (*hatched bars*). **(C)** The time of peak plasma LH levels in subjects treated with estrogen and progesterone. Numbers in parentheses are the number of subjects in each group. Each bar and vertical line indicate the mean and SE, respectively; *P < 0.05 vs. female control.

Representative changes in LH profiles over time are shown in Figure 1. The estrogen and progesterone treatment appeared to stimulate LH release in both female control and MTF subjects, but not in FTM subjects. The mean LH concentrations at each time point are shown in Figure 2A. We analyzed the %change in LH secretion. This analysis showed that the LH response in FTM subjects was significantly smaller than in female controls (Figure 2B, ANOVA *P* < 0.05, Fisher’s LSD *P* < 0.05). On the other hand, the LH response in MTF subjects was not different from that observed in female controls (Fisher’s LSD *P* > 0.5). The mean peak time of LH secretion in FTM subjects was significantly delayed compared to that observed in female controls (Figure 2C, Kruskal–Wallis *P* < 0.01, Dunn’s multiple comparison *P* < 0.05). The mean peak time of LH secretion in MTF subjects was not different from that observed in female controls (Dunn’s multiple comparison, no significant difference).

## Discussion

In the present study, FTM subjects were attracted to women, but the MTF subjects were attracted to women, men, or both sexes. This result was in good accord with previous reports (57–59). For example, Auer et al. (59) described an MTF group that comprised individuals attracted to women (gynephilic, 51%) or men (androphilic, 26%), and an FTM group that were attracted to women (gynephilic, 73%). These similarities to our data suggested that our sampling population was not biased.

By examining the LH response to estrogen combined with progesterone, we found that the neuroendocrine response was different between FTM subjects and female controls. We should consider the physiological meaning of this phenomenon. Progesterone is certainly an important factor for induction of the LH surge in the rodent (1, 60), the ewe (61), and the primate (47, 48). However, the physiological role of progesterone in the ovulation-induced LH surge has not been fully understood (62). For example, a primary question is, what is the source of progesterone? Progesterone in the follicular fluid in the ovary exhibits higher than in the blood of pregnant women and thus source of progesterone may be follicle cells (62). Progesterone may come from the central nervous system (63). Basically, in the menstrual cycle, a gonadotropin surge and follicular rupture occur, followed by the luteinization of granulosa cells. Granulosa cells then establish the corpus luteum and progesterone secretion increases. Thus, we speculated that, with the current understanding, it might be difficult to distinguish FTM subjects from control subjects, based solely on the LH response to estrogen, and suggest the importance of determining the LH controlling mechanism by means of progesterone treatment in transsexuals.

The majority of FTM subjects exhibited a regular menstrual cycle. Consequently, like female control subjects, they could reproduce the menstrual cycle, as shown by Gooren (24). We confirmed that some FTM subjects displayed a preovulatory LH surge (Funabashi and Kimura, unpublished observations), but we did not examine ovulation directly in this study. However, our previous studies suggested that there was a discrepancy in reproductive abilities; female rats exposed to a low dose of testosterone during the neonatal period displayed a surge-like LH secretion in response to the combination of estrogen and progesterone, but the number of gonadotropin-releasing hormone neurons that expressed Fos was attenuated (54). Since these rats exhibit a regular estrous cycle, these results are consistent with FTM in this study having a regular menstrual cycle, but exhibiting attenuated LH secretion in response to estrogen combined with progesterone. Thus, the discrepancy between the mechanism for controlling menstrual cycle and the steroid-induced LH surge may be reasonable but LH response by an exogenous stimulus is attenuated in FTM subjects.

We would like to discuss whether the LH controlling mechanism is different between the sexes in humans. A previous report found that this mechanism was not different between transsexual individuals and controls; therefore, they concluded that it was not sexually differentiated (24). In the present study, the majority of MTF subjects showed an increase in LH secretion in response to estrogen combined with progesterone, similar to female control subjects. This result suggested that the LH controlling mechanism was not sexually differentiated. This finding was supported by a previous report that suggested that a surge-like LH secretion could be induced in men by injecting progesterone after estrogen priming (50); however, that response was not compared with a response in women subjects in that report; therefore, sexual differentiation could not be ruled out. If it is reasonable to compare MTF subjects to FTM subjects, due to the opposite genetic backgrounds and sexual identities, different conclusions are drawn. Since LH responses in transsexuals were different, the alteration in sexual differentiation may be related to the GD etiology (25, 57). Thus, gender identity, sexual orientation, and reproduction might be sexually differentiated through different brain mechanisms. In previous studies, different brain areas were responsible for sexual orientation and gender identity; the suprachiasmatic nucleus and the bed nucleus of the stria terminalis, respectively (25, 28).

The main limitation of this study was the lack of a male control without testes, which prevented us from drawing conclusions. This study was also limited by the small number of GD subjects; thus, we lacked firm evidence to support our hypothesis. Consequently, our findings remain to be confirmed in future studies with larger numbers of subjects.

## Ethics Statement

This study was conducted in accordance with the recommendations of the Ethical Guidelines for Medical and Health Research Involving Human Subjects, established by the Ministry of Education, Culture, Sports, Science, and Technology and the Ministry of Health, Labor and Welfare. We obtained written informed consent to participate in the study from all volunteers, in accordance with the Declaration of Helsinki. The protocol was approved by the institutional Ethics Committee of the Yokohama City University School of Medicine. Subjects were interviewed to obtain data on their general health condition, sexual orientation, and menstrual cycles.

## Author Contributions

TF and FK designed the study, analysis and interpretation of data, and assisted in the preparation of the manuscript. TF wrote the initial draft of the manuscript. All other authors have contributed to data collection. FK critically reviewed the manuscript. All authors approved the final version of the manuscript, and agreed to be accountable for all aspects of the work in ensuring that questions related to the accuracy or integrity of any part of the work are appropriately investigated and resolved.

## Conflict of Interest Statement

The authors declare that the research was conducted in the absence of any commercial or financial relationships that could be construed as a potential conflict of interest.
